# EUS‐guided hydrogel microparticle injection in a cadaveric model

**DOI:** 10.1002/acm2.13266

**Published:** 2021-05-24

**Authors:** Seong‐Hun Kim, Kai Ding, Avani Rao, Jin He, Manoop S. Bhutani, Joseph M. Herman, Amol Narang, Eun Ji Shin

**Affiliations:** ^1^ Department of Internal Medicine Research Institute of Clinical Medicine of Jeonbuk National University‐Biomedical Research Institute of Jeonbuk National University Hospital Jeonju‐si Jeollabuk‐do Republic of Korea; ^2^ Department of Radiation Oncology and Molecular Radiation Sciences Johns Hopkins University Baltimore MD USA; ^3^ Department of Radiation Oncology University of Maryland Medical Center Baltimore MD USA; ^4^ Department of Surgery Johns Hopkins University Baltimore MD USA; ^5^ Department of Gastroenterology, Hepatology and Nutrition University of Texas MD Anderson Cancer Center Houston TX USA; ^6^ Radiation Oncology Zucker School of Medicine at Hofstra/Northwell Lake Success NY USA; ^7^ Division of Gastroenterology and Hepatology Johns Hopkins University Baltimore MD USA

**Keywords:** endosonography, hydrogel, pancreatic neoplasms, radiation, stereotactic body radiotherapy

## Abstract

**Background and Aims:**

A potential method to reduce gastrointestinal toxicity during radiation therapy in pancreatic head cancer is to create a physical space between the head of the pancreas (HOP) and the duodenum. To date, there have been early reports on the feasibility of endoscopic ultrasound (EUS)‐guided hydrogel injection into the interface between the HOP and the duodenum to increase the peri‐pancreatic space for radiotherapy. We aimed to evaluate the technical feasibility of EUS‐guided hydrogel injection for the creation of space at the peri‐pancreatic interface in a cadaveric model.

**Methods:**

Baseline abdominal computerized tomography (CT) was performed on three unfixed cadaveric specimens. The hydrogel was injected transduodenally into the interface between the HOP and duodenum using linear‐array EUS and a 19G needle for fine needle aspiration (FNA). This procedure was repeated along the length of the HOP. CT imaging and gross dissection were performed after the procedure to confirm the localization of the hydrogel and to measure the distance between the HOP and the duodenum.

**Results:**

All cadavers underwent successful EUS‐guided injection of the hydrogel. Cadavers 1, 2, and 3 were injected with 9.5, 27, and 10 cc of hydrogel, respectively; along the HOP, the formation of the peri‐pancreatic space was a maximum size of 11.77, 13.20, and 12.89 mm, respectively. The hydrogel injections were clearly visualized as hyperechoic bullae during EUS and on post‐procedure CT images without any artifacts in all cases.

**Conclusions:**

We demonstrated that EUS‐guided delivery of hydrogel is feasible, and that it increases the peri‐pancreatic space in a cadaveric model. The polyethylene glycol (PEG) hydrogel was clearly visible on EUS and CT, without significant artifacts. This may lead to new treatment approaches for pancreatic carcinomas.

## 1 INTRODUCTION

Pancreatic cancer has a very poor prognosis, with a 5‐yr survival rate of 8%, and is the third leading cause of cancer‐related deaths in the United States.^1^ Surgical resection is required for long‐term survival of patients with pancreatic cancer, but less than 20% of the diagnosed patients are eligible to undergo surgery.^2^ Patients who were staged at the time of diagnosis as borderline resectable (median survival, up to 20 months), locally advanced or unresectable (median survival, 8–14 months), and metastatic stage (median survival, 4–6 months) face difficulty in qualifying for surgery.^3^ Despite the poor prognosis in these patients, the primary tumors should be treated to increase the number of patients who can eventually undergo surgery and to slow down the complications resulting from the local progression of the primary tumor.^4^

One of the important treatment options for primary tumors is chemoradiotherapy (CRT).^4^ The role of radiotherapy (RT) in locally advanced pancreatic cancer (LAPC) is still under debated, but in patients who respond to chemotherapy early on in the treatment, CRT is one of the best ways to reduce the rate of local progression.^5^, ^6^, ^7^ Recently, a steady improvement in RT has enabled higher dose delivery to the primary site and increased the effectiveness of chemotherapy.^8^ Among these methods, stereotactic body radiotherapy (SBRT) increases the therapeutic effect by effectively delivering a higher dose of radiation than the conventional single dose.^4^, ^6^ Specifically, SBRT has two advantages: reducing the duration of RT treatment and preventing breaks in chemotherapy.^4^, ^9^ However, SBRT has the disadvantage of increasing acute and late gastrointestinal (GI) toxicity in adjacent organs such as the duodenum and stomach.^6^ Accordingly, various efforts have been made to reduce the GI toxicity of SBRT. For example, attempts have been made to reduce the planning target volume (PTV) or deliver radiation during the breathing cycle.^10^ However, reports on the effectiveness of these previous methods are limited.

Recently, in order to reduce GI toxicity and deliver a more effective radiation dose to the primary tumor, biomaterials have been injected between the GI wall and primary tumors to create a space for separation.^11^ Until now, most of the studies have focused on RT for prostate cancer, with no clinical studies on pancreatic cancer.^11^, ^12^ A good spacer requires the following: First, insertion should be easy. Second, complications should be minimal, enough to be accepted. Third, it should be stable after the insertion. Fourth, it should be visible upon imaging. Finally, it should be naturally degraded after the treatment is finished.^13^ Currently available biomaterials include blood patches, hyaluronic acid, and collagen.^11^, ^14^ However, these biomaterials have a short durability, unreliable degradation, and uneven distribution during RT treatment.^14^ Recently, a novel injectable hydrogel, synthesized as iodinated polyethylene glycol (PEG) hydrogel microparticles, has attracted attention as a new spacer.^15^ PEG hydrogel has several advantages over other biomaterials as characteristics include water solubility, high mobility in solution, lack of toxicity, lack of immunogenicity, and reliable excretion from the body.^16^ This new injectable PEG hydrogel has been approved by the US Food and Drug Administration (FDA) as a soft tissue fiducial marker and has been known to be stable *in vivo* for 3 months, absorbed at 7 months, and excreted through renal filtration.^17^ It was also stable and efficacious as a rectal spacer in the Prostate Cancer Phase III trial.^18^ Anonymous 2017 reported the possibility of PEG hydrogel use and safety in pancreatic cancer radiation therapy in the cadaveric model and porcine model.^19^, ^20^ However, only a few cases have been directly applied to humans. The standardization of techniques using endoscopic ultrasound (EUS), which has been widely used in recent years, has not been established. We aimed to evaluate whether it is technically feasible to inject a EUS‐guided hydrogel between the pancreatic head and duodenum wall in a cadaveric model.

## 2 MATERIALS AND METHODS

### 2.A Cadaveric specimens

Three cadaveric specimens (refrigerated, unfixed, unfrozen, and deidentified) were used within the first 3 days postmortem. This study was conducted in accordance with approval by the institutional review board of the authors’ affiliated institution.

### 2.B PEG hydrogel

**Fig. 1:**
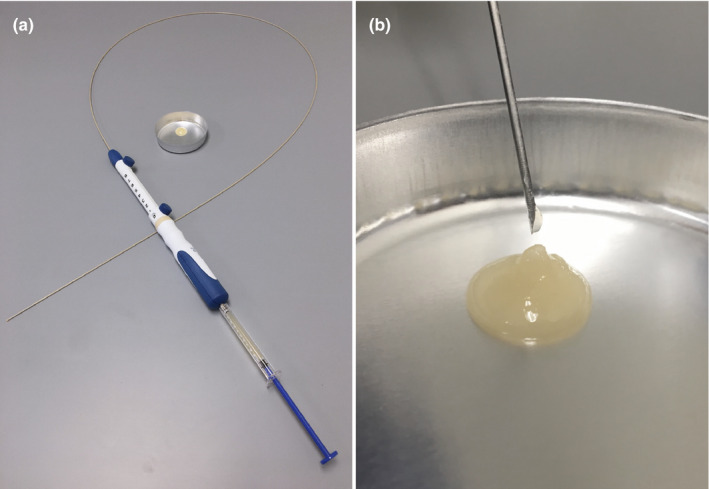
Polyethylene glycol (PEG) hydrogel and needle. (a) The plastic receiving syringe mounted on standard 19‐gauge for fine needle aspiration needle. (b) High viscosity PEG hydrogel.

A novel injectable hydrogel, synthesized as iodinated PEG hydrogel microparticles (TraceIT Tissue Marker; Augemenix, Waltham, MA, USA) was used. PEG is an absorbable tissue marker that was approved by the US FDA in 2013 and was approved as a fiducial marker and gel system by the European Conformity (CE) Mark in the same year.^21^ The TraceIT Tissue Marker consisted of a glass pre‐filled syringe, a plastic receiving syringe, a sterilized luer‐luer connector, and a needle. The PEG hydrogel was mixed immediately before use. After mixing five times between the two syringes, an injectable PEG hydrogel was placed in a plastic receiving syringe (Fig. 1).^21^

### 2.C EUS‐guided PEG hydrogel injection

**Fig. 2:**
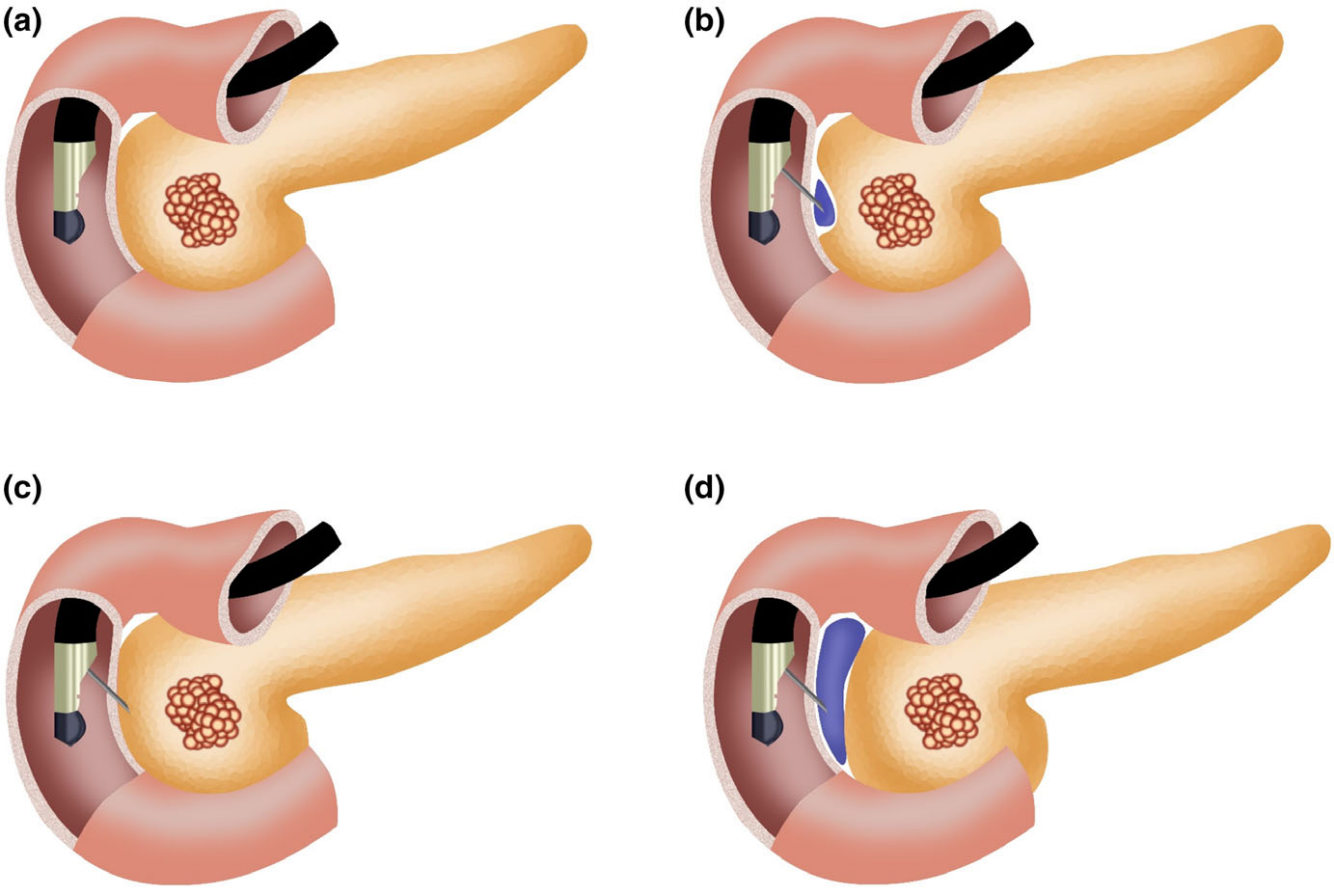
EUS‐guided polyethylene glycol (PEG) hydrogel injection. (a) EUS endoscope in the duodenum to identify the pancreatico‐duodenal interface for PEG hydrogel insertion. (b) Insertion of a standard for fine needle aspiration needle into the pancreatic head margin. (c) A small amount of PEG hydrogel is injected into the interface between the pancreatic head and duodenal wall. (d) The needle is retracted slightly and placed securely in the interface to inject the PEG hydrogel until there is an adequate bullae.

Before the intervention, a baseline CT (Philips Brilliance Big Bore CT, 2‐mm slices, 120 kVp, 200 mA, and 60‐cm field of view) was performed on each of the three unfixed cadaveric specimens. Using a linear‐array EUS scope (Pentax EG‐3870UTK; Pentax Europe GmbH, Hamburg, Germany) and ultrasound equipment unit (Hitachi‐Preirus US platform, Hitachi Medical Corp., Tokyo, Japan), we identified the space between the duodenal wall and the head of the pancreas in a cadaveric model [Fig. 2(a)]. Then, we injected the PEG hydrogel into the peri‐pancreatic space by creating a visible separation between the duodenal wall and the pancreatic parenchyma using a standard 19‐gauge needle for fine needle aspiration (FNA) [Fig. 2(b)]. If there was not enough space in the peri‐pancreatic space during the initial PEG hydrogel injection, a small amount was first injected into the boundary of the pancreatic parenchyma with withdrawal of the needle until the space was identified and created [Fig. 2(c)]. If the space was readily observed in the real‐time EUS image with the needle located in the space, the PEG hydrogel was injected until sufficient bullae were formed [Fig. 2(d)]. The needle was then removed from the duodenal lumen. The procedure was repeated along the length of the head and uncinate of the pancreas. All procedures were performed by a skilled endoscopist. CT was performed immediately after the procedure to confirm the location and to measure the distance between the duodenum and the pancreas. Gross dissection of the pancreas and duodenum was performed by a pancreatobiliary surgeon after CT to grossly evaluate localization and the effect of the PEG hydrogel on the tissue.

### 2.D Planning methods

SBRT simulation CT scans of the cadaveric specimens before and after injection of the PEG hydrogel were acquired with a 2 mm slice thickness (Philips Brilliance Big Bore CT, 120 kVp, 200 mA, 60‐cm field of view). The CT scans were imported into a radiation therapy planning system (Pinnacle; Philips Radiation Oncology Systems, Milpitas, CA) to evaluate the effect of dose sparing from the injected PEG hydrogel. Virtual tumors of 2 cm diameter were created in the head of the pancreas in the simulation CT scans as gross tumor volumes (GTVs). For SBRT treatment preparation, PTVs were created with the expansion of a 2‐mm margin from the GTVs. Organs at risk (OARs) including the proximal duodenum, small bowel liver, kidneys, spinal cord, and stomach were contoured. Following our institutional SBRT protocol with a prescription dose of 33 Gy in five fractions, we set dose constraints for the proximal gastrointestinal OARs with V15 Gy (volume receiving 15 Gy or more) as follows: <9 cc, V20 Gy < 3 cc, and V33 Gy < 1 cc. We also set the constraints for the other OARs with liver V12 <50%, combined kidney V12 < 75%, and spinal cord V8 < 1 cc. Multiple beams (10 or 11), with a direct machine parameter optimization algorithm, were used to generate the SBRT plans, as previously described.^22^

## 3 RESULTS

**Fig. 3:**
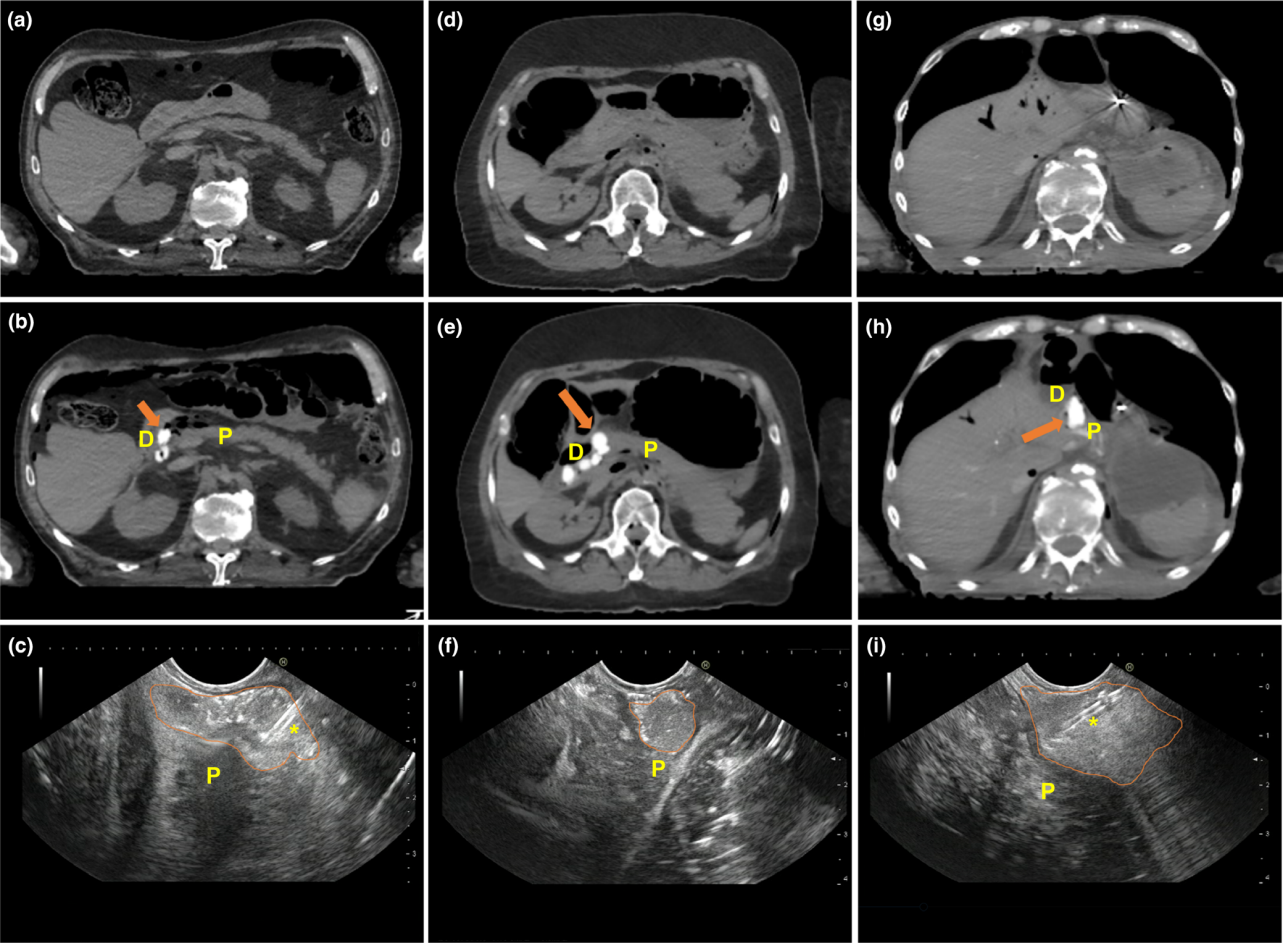
Pre‐ and post‐CT and endoscopic ultrasound (EUS) images of polyethylene glycol (PEG) hydrogel injection. (a) Pre‐injection CT on Cadaver 1. (b) Post‐injection CT on Cadaver 1, the orange arrow indicates injected PEG hydrogel. (c) EUS imaging during PEG hydrogel injection on Cadaver 1, the orange region delineates injected PEG hydrogel. (d) Pre‐injection CT on Cadaver 2. (e) Post‐injection CT on Cadaver 2, the orange arrow indicates injected PEG hydrogel. (f) EUS imaging during PEG hydrogel injection on Cadaver 2, the orange region delineates injected PEG hydrogel. (g) Pre‐injection CT on Cadaver 3. (h) Post‐injection CT on Cadaver 3, the orange arrow indicates injected PEG hydrogel. (i) EUS imaging during PEG hydrogel injection on Cadaver 3, the orange region delineates injected PEG hydrogel. (Abbreviations; P, Pancreas, D, Duodenum, Asterisk, 19G FNA needle.).

**Fig. 4:**
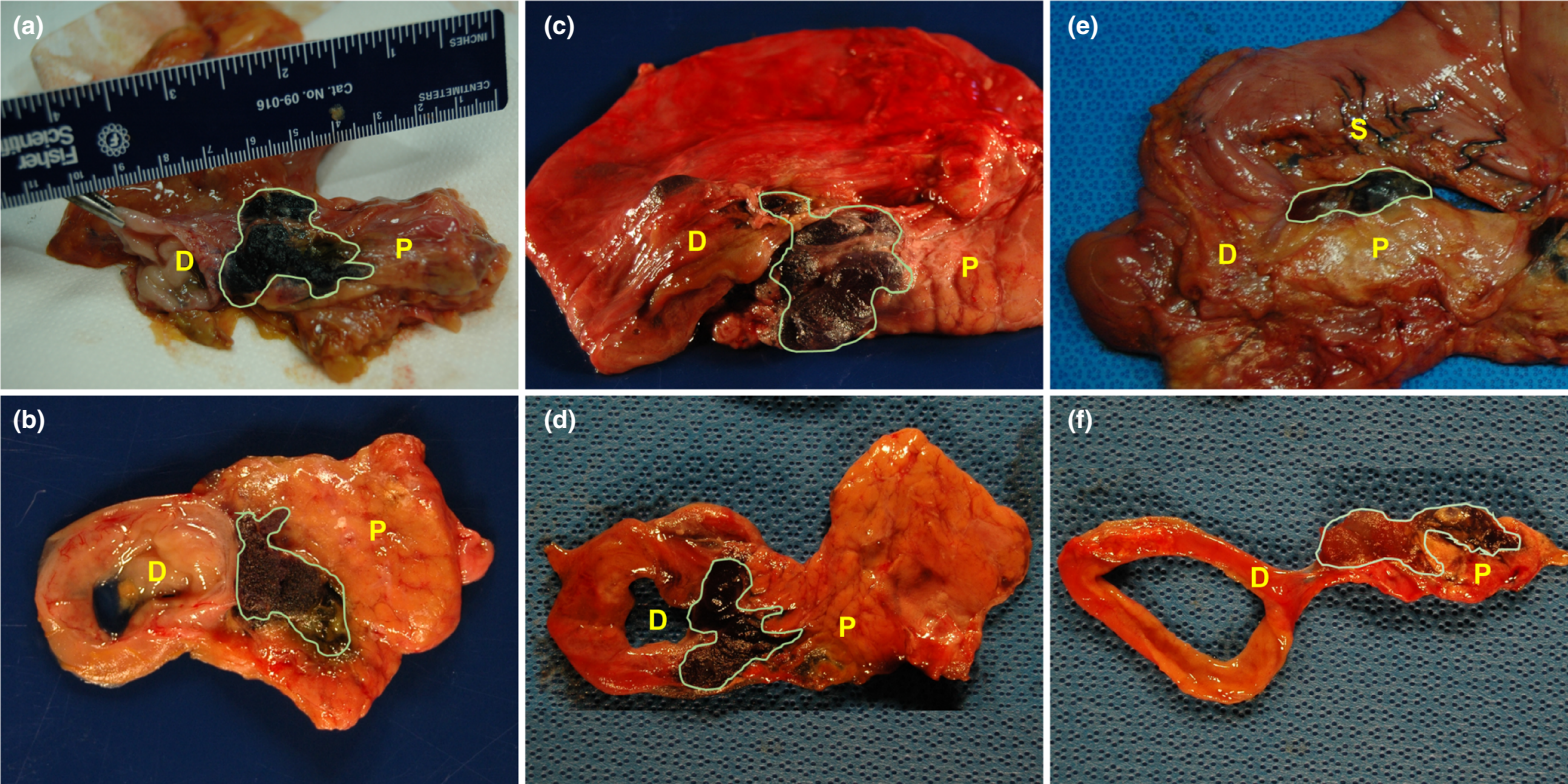
Gross histology pictures after polyethylene glycol (PEG) hydrogel injection. (a & b) Cadaver 1. (c & d) Cadaver 2. (e & f) Cadaver 3. The dissection and formation of the new space between the pancreatic head and the duodenal wall was well observed after PEG hydrogel injection in all cadaveric models. Green area delineates injected PEG hydrogel. (Abbreviations; P, Pancreas, D, Duodenum, S, Stomach.).

**Fig. 5:**
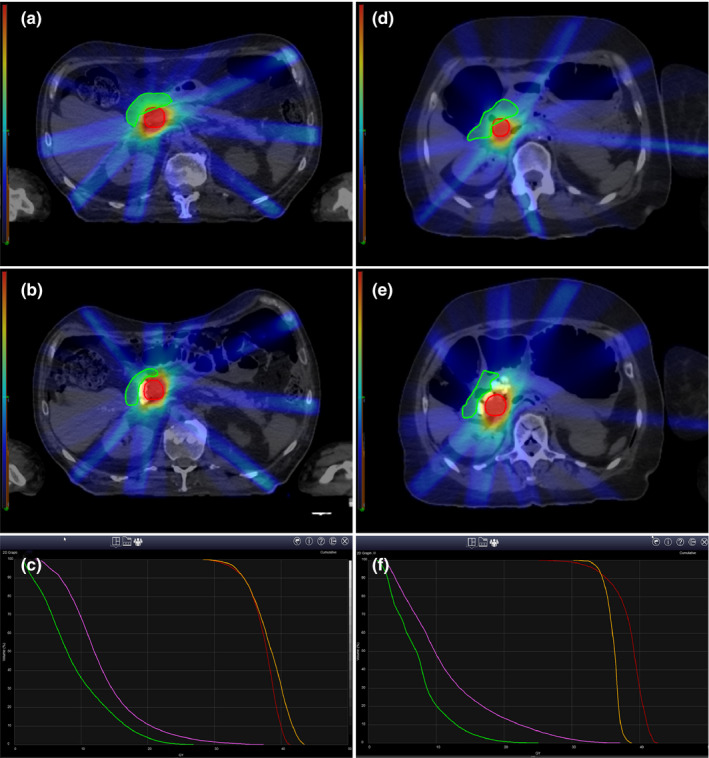
Comparison of planning target volume (PTV) on simulation planning CT according to stereotactic body radiotherapy (SBRT) virtual treatment plan before and after polyethylene glycol (PEG) hydrogel injection. (a & b & c) Cadaver 1. (d & e & f) Cadaver 2. (a) Pre‐injection simulation CT on Cadaver 1. (b) Post‐injection simulation CT on Cadaver 1. (d) Pre‐injection simulation CT on Cadaver 2. (e) Post‐injection simulation CT on Cadaver 2. In simulated CT before injection of PEG hydrogel, the PTV of the duodenum and the pancreas region seem to overlap, but after the PEG hydrogel injection, there is no overlap between the PTV of the duodenum and the pancreas region on the simulation CT. (Green area denotes duodenum, Red area denotes pancreas) (c & d) Dose‐volume histograms show the changes in the proximal duodenum overlap volume histogram metric (pre‐spacer proximal duodenum [pink line], post‐spacer proximal duodenum [green line]), and PTV coverage placement (pre‐spacer PTV [red line] and post‐spacer PTV [orange line]).

All three cadavers underwent a successful EUS‐guided injection of the PEG hydrogel. An overview of the cadaveric specimens and the technical results are described in Table 1. In Cadaver 1, a total volume of 9.5 cc was injected along the interface between the head of the pancreas and duodenum, and the maximum diameter of the new space formed between the pancreas and the duodenum was 11.77 mm [Figs. 3(a)–3(c)]. In Cadaver 2, a total volume of 27 cc was injected along the interface between the head of the pancreas and duodenum, the maximum diameter of the new space formed between the head of the pancreas and the duodenum was 13.2 mm [Figs. 3(d)–3(f)]. In Cadaver 3, a total volume of 10 cc was injected along the interface between the head of the pancreas and duodenum, the maximum diameter of the new space formed between the pancreas and the duodenum was 12.89 mm [Figs. 3(g)–3(i)]. In all three cases, the PEG hydrogel was well observed by the hyperechogenic appearance of the hydrogel on EUS during the procedure, and the localization of the hydrogel injection was well observed in real time [Figs. 3(c), 3(f), and 3(i)]. In addition, the injected hydrogel was well observed due to its hyperechogenicity on post‐procedure CT without artifacts in all cases [Figs. 3(b), 3(e), and 3(h)]. After injection of the PEG hydrogel, all cadavers were examined. Pre‐injection CT and post‐injection CT varied substantially regarding anatomy (bowel regions and air bubbles). However, the variation was not caused by hydrogel injection. During the EUS, air enters the intestine through the scope. Therefore, the anatomy shown by pre‐injection CT and post‐injection CT may appear different. The dissection and formation of a new space between the pancreatic head and duodenal wall were confirmed by gross histology. The injected PEG hydrogel was well visualized as a blue gel (Fig. 4). SBRT plans on the simulation CT scans before and after PEG injection were evaluated for dosimetric analysis. Simulation CT scans before the injection of the PEG hydrogel, showed that the proximal duodenum overlapped the PTV regions [Fig. 5(a) and 5(d)]. This is because of the 2‐mm margin of expansion from the GTV to the PTV. Due to the overlap between the PTV and proximal duodenum, PTV coverage could not be achieved without violating the dose constraint of the proximal duodenum. The proximal duodenum V20 values were 3.86 and 3.75 cc for Cadaver 1 and Cadaver 2, respectively, both violating the constraint of 3 cc as described previously. In Cadaver 2, the proximal duodenum V15 value was 9.12 cc, which also violated the V15 dose constraint. According to simulation CT scans after injection of the PEG hydrogel, there was no overlap between the PTV and the proximal duodenum [Fig. 5(b) and 5(e)]. The PEG hydrogel injected between the pancreatic head and duodenum increased the separation between the PTV and the proximal duodenum. As a result, both the SBRT plans of the scans after PEG hydrogel injection met the dose constraints of the proximal duodenum. For Cadaver 1, the proximal duodenum V20 decreased by 90.7% (3.86 cc vs 0.36 cc) and V15 decreased by 71.4% (7.07 cc vs 2.02 cc). For Cadaver 2, the proximal duodenum V20 decreased by 71.2% (3.75 cc vs 1.08 cc) and V15 decreased by 57.1% (9.12 cc vs 3.91 cc) [Figs. 5(c) and 5(f)].

**Table 1:** An overview of cadaveric specimens and technical results.

	Syringe/Size of FNA Needle	Total volume on injection	Maximal diameter of new space along HOP	Ease of injection	Localization of PEG	CT visibility	EUS visibility
Cadaver 1	1mL/ 19G	9.5 mL	11.77 mm	Easy	Good	Excellent	Good
Cadaver 2	1mL/ 19G	27.0 mL	13.20 mm	Easy	Good	Excellent	Good
Cadaver 3	1mL/ 19G	10 mL	12.98 mm	Easy	Good	Excellent	Good

Abbreviation: HOP, head of pancreas.

## 4 DISCUSSION

Until now, there have been only early studies on the technical feasibility of PEG hydrogels in the pancreaticoduodenal space. Anonymous 2017 focused predominantly on the radiation oncology aspect in terms of dosimetric and physical characteristics.^19^, ^20^ Thus, this study was conducted in a cadaveric model and is the first attempt to show whether injecting PEG hydrogel between the pancreatic head and the duodenum is technically feasible. According to the results of the study, the novel PEG gel was clearly visualized during real‐time EUS and was easily seen without artifacts as per post‐procedure simulation CT. In addition, pathological evaluation revealed good separation between the pancreatic head and duodenum. Using simulation CT for the planning of virtual radiotherapy, the overlapping area of the pancreatic head and the duodenal wall after PEG gel injection was clearly reduced, and the duodenal PTV was also clearly reduced. To date, PEG hydrogel injection via EUS has been reported as a liquid fiducial marker in esophageal cancer, and two cases have reported use in pancreatic cancer as an abstract only as a liquid fiducial marker.^23^, ^24^, ^25^ However, the role of PEG gel as a spacer via EUS injection and its feasibility in pancreatic cancer are unknown.

To date, biomaterial spacers have been attempted to reduce GI wall toxic side effects during RT, predominantly in prostate cancer, including blood patch, hyaluronic acid, collagen, and PEG hydrogel.^11^, ^14^, ^26^ The blood patch is injected between the rectum and the prostate to reduce the radiation dose on the rectal wall. However, there were only three cases in the study, and only 1 week of follow‐up was performed, rendering it difficult to evaluate long‐term stability and clinical effectiveness.^27^ Hyaluronic acid (HA) is a glycosaminoglycan‐based polymer present in human connective tissue and the extracellular matrix, and has a high affinity for water, similar to PEG hydrogel.^28^ At present, HA has been used in clinical applications in two forms, one as a natural modification and the other that is fully synthesized.^26^ There are several reports on the role of HA in spacer treatment in the treatment of prostate cancer.^26^ HA is also known to be absorbed through the liver and kidneys 6–12 months after injection.^26^, ^28^ Unlike PEG hydrogels, however, exposure to radiation accelerates the absorption period to 4–8 months.^29^ Although it may not be a significant problem considering the duration of radiation therapy in pancreatic cancer, this instability makes it difficult to predict the absorption period after therapeutic radiation exposure, which may be a disadvantage.^29^ Moreover, the viscosity of HA is also known to be higher than that of PEG gel. For this reason, 16G or 17G have been used for HA injections in previous prostate cancer studies.^29^, ^30^ However, in the case of pancreatic cancer, HA injections may prove to be technically challenging due to the need to use thinner and longer needles through EUS compared to the needles used in the case of prostate cancer. Collagen is the most abundant protein in the human body, is a major component of connective tissue, and is widely used in clinical practice because of its structural stability and stretch resistance in many tissues.^26^ However, since collagen is volatile in nature, it is supplied in lyophilized form and tends to aggregate during preparation for injection, which may make it difficult to use in clinical practice; only one clinical study of collagen, as a spacer, has been reported thus far.^14^, ^31^

PEG hydrogels have a net‐like structure of PEG oligomers and can safely contain large amounts of water. As a result, PEG gels are a stable and flexible gel form.^26^ PEG hydrogels have several advantages: First, the PEG hydrogel was dimensionally stable for 3 months, followed by rapid disappearance. In comparison, HA is enzymatic and shrinks over time.^15^, ^32^, ^33^ This enables the physician to see the critical interface between the PTV and the OARs throughout RT, which has benefits for localization, tracking, and alignment. Second, the PEG hydrogel had a predictable absorption rate (excreted by 7 months).^15^, ^32^ Third, PEG molecules are unchanged when exposed to therapeutic radiation, whereas HA molecules are lysed, resulting in an increased absorption rate.^34^, ^35^ Fourth, PEG hydrogel has a lower viscosity than HA, enabling fine needle injections via EUS FNA needle. In the prostate cancer study, an 18G needle was used to inject the PEG hydrogel compared to a 16‐17G needle needed for HA injection.^13^, ^14^, ^26^, ^29^, ^32^ Also, in the case of thoracic and esophageal malignancies a 22G needle was used.^17^, ^24^ The PEG hydrogel must be injected at a faster rate than HA and collagen because the substance solidifies in approximately 10 s after exposure to water. However, the low viscosity compared to HA and collagen at the first injection provides the advantage of ease of injection within the needle and can be used in relatively thin and long needles like the EUS‐FNA needle.^26^, ^35^ Fifth, PEG hydrogel is synthetic and proven to be bacteriostatic, suggesting that the risk of infection is low and theoretically, there is a very low possibility of an immunological reaction.^26^

This is the first study to use TracelT as a spacer in the pancreas. The PEG hydrogel used in the previous prostate cancer study was SpaceOAR (SpaceOAR System, Augmenix, Inc., Waltham, MA). There are several technical reasons why we did not use SpaceOAR. First, the hydrogel in the SpaceOAR system must be injected simultaneously. Otherwise, the delivery system will clog, making further injections impossible. This property may result in an uneven gel distribution between the pancreas and the duodenal wall after injection.

The best way to inject a PEG hydrogel between the pancreatic head and duodenum is by using the standard FNA needle under EUS guidance. In pancreatic disease, EUS allows real‐time monitoring of pancreatic lesions, and the safety and efficacy of FNA needle use are well known.^36^ These advances in technology have enabled various therapeutic interventions for pancreatic lesions.^37^ The technique of injecting PEG hydrogel through EUS between the pancreatic head and duodenum is not significantly different from that of conventional EUS‐guided interventions. However, the PEG hydrogel injection technique differs from existing EUS interventions as follows. First, the PEG hydrogel should be observed in real time using EUS. In this study, a novel injectable hydrogel, synthesized as iodinated PEG hydrogel microparticles, was easily visualized on EUS due to its hyperechoic appearance. Second, the viscosity of the liquid spacer is very important in pancreatic applications because the needle available for EUS is thinner (19–25 gauge) and longer (working length: 137.5 cm to 141.5 cm), compared to the needles used in the previous prostate cancer study.^38^ The PEG hydrogel used in this study was easily injected through a standard 19G FNA needle. Third, the pancreatic head is closely adherent to the duodenum without a visible space between them on real‐time EUS or other imaging studies, but there is a potential space. Therefore, we started to inject at the pancreatic head margin first, and when there was a small space between the pancreatic head and the duodenum, the needle was pulled back slightly to securely place it at the interface. Although PEG hydrogel was used as a fiducial marker in pancreatic cancer in a previous study, the effect of PEG hydrogel on pancreatic tissue was poorly studied.^23^ Fourth, the inserted spacer should be stable during RT. It is possible to predict that the hydrogel injection is stable even in the vicinity of the pancreatic head because it was previously shown to be stable in a study using TraceIt in esophageal cancer, where mediastinal structures are associated with significant physiologic movements. In this study, it was confirmed that the PEG hydrogel injections were stable, as pancreatic head and duodenal dissection were performed visually and microscopically post‐procedure.^25^

This study has limitations in that the cadaver model was used, and the number of cases was small. Notably, the structures around the pancreas of the cadaver model and the human are clearly different. It is difficult to distinguish blood vessels in cadavers on EUS because there is no flow in the vessels of the phantom. In addition, the duodenum wall of the cadaver model was also thinner than in humans, so it was difficult to find the plane to initiate the hydrogel injection. In addition, there is also no breathing/motion issue for cadavers. Although the area around the pancreas is more complex than the prostate, EUS allows real‐time monitoring of pancreatic lesions, and the safety and efficacy of FNA needle use are well known. The technique of injecting PEG hydrogel through EUS between the pancreatic head and duodenum is not significantly different from that of conventional EUS‐guided injections. In addition, in general, when EUS is performed, the movement of the walls of the pancreas and duodenum is relatively small. Moreover, as the duodenal wall tissue is viable in real patients, the procedure may be technically easier in real patients than in a cadaver. Another limitation is that we could not quantify the increase in the pancreaticoduodenal space in the three dimensions after PEG injection. Therefore, further studies are needed to evaluate the potential clinical applications of PEG hydrogel injections. First, it is necessary to confirm that the PEG hydrogel can be inserted through a thinner 22G needle. If possible, using a thinner needle will make the procedure technically easier in the duodenum and potentially less likely to cause complications such as aborted procedures, perforation, and bleeding. Second, more research is needed to determine the effect of PEG hydrogel on the pancreatic parenchyma and duodenal wall. Theoretically, it should be easier to find the space between the pancreatic head and duodenum in a clinical model than in a cadaver model. However, given the high likelihood that a portion of the spacer will be injected into the pancreatic parenchyma and duodenal wall, further studies are needed to evaluate its safety and the potential for causing other complications such as inflammation, compression necrosis, or the like in the tissue. Third, it is necessary to investigate whether the injected PEG hydrogel can be maintained in a stable position in the space between the pancreatic head and duodenum during RT. Lastly, the injected hydrogel volume is decided during the procedure. During the EUS guided hydrogel injection, the injection volume or the number of blebs increases until the spacing requirement is achieved. To reduce OARs, the minimum amount of hydrogel required, should be discussed with the radio‐oncologist, and a more well‐designed overlap volume prediction model, to predict the hydrogel spacing required to achieve clinical constraints, is needed in the future.

## 5 CONCLUSIONS

We demonstrated that EUS‐guided delivery of hydrogel is feasible, and that it increases the peri‐pancreatic space in a cadaveric model. The PEG hydrogel was clearly visualized on EUS and CT, without significant artifacts. This EUS‐guided technique of injecting PEG hydrogel between pancreatic head tumors and the duodenum to separate the duodenum from the tumor for higher radiation dosage while protecting the duodenum may open up new paradigms for radiotherapy in pancreatic cancer. The self‐absorbing capacity of the hydrogel in 3 months provides sufficient time for effective radiation delivery and recovery, thereby increasing its attractiveness for clinical application. Further studies are warranted to evaluate the feasibility, effectiveness, and safety of clinical models.

## AUTHOR CONTRIBUTION

Study concept and design: S.K., K.D., A.R., A.N., and E.S. Acquisition of data analysis and interpretation of data: S.K., K.D., A.R., J.H., A.N., and E.S. Drafting of the manuscript: S.K., K.D., and E.S. Critical revision of the manuscript for important intellectual content: A.R., J.H., M.S.B., J.M.H., and A.N. Supervision: E.S. All the authors have approved the final version of the manuscript and agree to be accountable for all aspects of the work in ensuring that questions related to the accuracy or integrity of any part of the work are appropriately investigated and resolved.

## CONFLICT OF INTEREST

Kai Ding was supported by the Augmenix research. Manoop S. Bhutani was supported by OncoSil‐research, Galera‐research, Augmenix‐research, and Silenseed‐research support. Joseph M. Herman was supported by Augmenix research, Medtronic consultant, and 1440 foundation. Amol Narang supported the Augmenix research. Eun Ji Shin was supported by the Boston Scientific Consultant and Medtronic consultant. The other authors declare that they have no conflicts of interest.

## ETHICAL APPROVAL

This study was conducted in December 2016, according to the institutional review board approval by John Hopkins Hospitals (CIR00023988).

## Data Availability

The data that support the findings of this study are available from the corresponding author upon reasonable request.
